# New-Onset and Relapsed Membranous Nephropathy post SARS-CoV-2 and COVID-19 Vaccination

**DOI:** 10.3390/v14102143

**Published:** 2022-09-28

**Authors:** Qiqi Ma, Xiang Li, Gaosi Xu

**Affiliations:** Department of Nephrology, The Second Affiliated Hospital of Nanchang University, No. 1, Minde Road, Donghu District, Nanchang 330006, China

**Keywords:** membranous nephropathy, SARS-CoV-2, COVID-19, vaccination

## Abstract

Since the severe acute respiratory syndrome coronavirus 2 (SARS-CoV-2) outbreak and COVID-19 vaccination, new-onset and relapsed clinical cases of membranous nephropathy (MN) have been reported. However, their clinical characteristics and pathogenesis remained unclear. In this article, we collected five cases of MN associated with SARS-CoV-2 infection and 37 related to COVID-19 vaccination. Of these five cases, four (4/5, 80%) had acute kidney injury (AKI) at disease onset. Phospholipase A2 receptor (PLA2R) in kidney tissue was negative in three (3/5, 60%) patients, and no deposition of virus particles was measured among all patients. Conventional immunosuppressive drugs could induce disease remission. The underlying pathogenesis included the subepithelial deposition of viral antigens and aberrant immune response. New-onset and relapsed MN after COVID-19 vaccination generally occurred within two weeks after the second dose of vaccine. Almost 27% of patients (10/37) suffered from AKI. In total, 11 of 14 cases showed positive for PLA2R, and 20 of 26 (76.9%) presented with an elevated serum phospholipase A2 receptor antibody (PLA2R-Ab), in which 8 cases exceeded 50 RU/mL. Conventional immunosuppressive medications combined with rituximab were found more beneficial to disease remission for relapsed patients. In contrast, new-onset patients responded to conservative treatment. Overall, most patients (24/37, 64.9%) had a favorable prognosis. Cross immunity and enhanced immune response might contribute to explaining the mechanisms of MN post COVID-19 vaccination.

## 1. Introduction

The ongoing pandemic of coronavirus disease 2019 (COVID-19) caused by severe acute respiratory syndrome coronavirus 2 (SARS-CoV-2) has brought significant challenges to human beings. As of 11 September 2022, more than 605 million infected cases have been confirmed globally, and deaths have reached 6.4 million [1]. Current research found that SARS-CoV-2 could affect multiple human organs with a high expression of angiotensin-converting-enzyme 2 (ACE2) receptor, including respiratory tracts, heart, kidney, nervous system, etc. [2].

Admittedly, the rapid development of vaccines effectively curbed COVID-19 prevalence and transmission, including mRNA vaccines (Pfizer-BioNTech, America; Moderna, America), inactivated vaccines (Sinovac Life Sciences, China), and adenovirus vector vaccines (AstraZeneca, America; Johnson & Johnson, America), all of which relied upon and aimed to present spike protein to the immune system despite different functional patterns. Common adverse reactions after COVID-19 vaccination included fever, headache, fatigue, myalgia, etc. [3]. Nevertheless, since massive vaccination, growing numbers of clinical cases concerning glomerular diseases such as membranous nephropathy (MN) [4], IgA nephropathy [5,6], and minimal change disease [7], etc., have been widely reported.

MN was an autoimmune glomerular disease in adults, usually manifested as edema and proteinuria. Approximately one-third of patients could achieve spontaneous remission [8], and immune therapies were considered to be the preferred treatment regime, including B lymphocyte depletion, steroids, cyclophosphamide (CTX), and calcineurin inhibitors [8]. With the continuous increase of infected individuals and the universal application of COVID-19 vaccination, multiple clinical cases of new-onset and relapsed MN were reported, whereas the association between them remained mysterious. In this review, we systematically summarized the clinical features of new-onset and relapsed MN post SARS-CoV-2 infection and COVID-19 vaccination, elaborated their treatment and prognosis, and first proposed several potential mechanisms of the disease.

## 2. New-Onset MN post SARS-CoV-2 Infection

### 2.1. Clinical Features and Follow-Up

We performed a literature review via searching the electronic database, including PubMed, EMBASE, Google Scholar, and Web of Science, taking (“membranous nephropathy” OR “proteinuria” OR “nephrotic syndrome”) AND (“SARS-CoV-2” OR “COVID-19” OR “2019-ncov” OR “novel coronavirus” OR “coronavirus”) as the keywords to acquire the clinical cases of MN related to SARS-CoV-2 infection.

In total, five cases of MN associated with SARS-CoV-2 infection have been identified before 6 September 2022 in four articles [9,10,11,12] (Table 1)—all new, including one female and four males. Four elderly cases had a previous chronic medical history, especially hypertension. Three cases developed edema, and one case showed massive proteinuria. All patients underwent kidney biopsy (phospholipase A2 receptor (PLA2R)—two positive, three negative; viral particles, all negative). Only one case [11] presented with PLA2R-Ab positive expression. The auxiliary examination of four cases suggested acute kidney injury (AKI) at admission.

One elderly female case [9] saw improvement in edema without any intervention, and the other case [9] responded to tacrolimus (TAC), but his follow-up records were not available. One male case [10] died of worsened respiratory status within 16 days. Despite that, the albumin increased from 17 to 26 g/L, and serum creatinine decreased from 7.1 to 3.7 mg/dL with the treatment of lenzilumab and intravenous methylprednisolone. A young patient [11] initially received angiotensin-converting-enzyme inhibitor (ACEI), whereas there was no remission at 3 months, followed by CTX combined with prednisolone which achieved gradual remission within 2 months. One elderly male case [12] had been dependent on dialysis for 80 days.

### 2.2. Treatment and Prognosis

Case reports on new-onset MN post SARS-CoV-2 infection were rare. Only one case [9] achieved spontaneous remission without treatment. Steroids, CTX, and TAC were the principal immunotherapy approaches. Up to now, the published cases of MN associated with SARS-CoV-2 infection were not recommended using rituximab (RTX) (Table 1, case 1–5). Previous literature reported that RTX treatment could cause viral reactivation among patients with hepatitis B virus-associated MN [14]. Whether this phenomenon would occur in MN post SARS-CoV-2 infection still needs further clarification. In contrast, the prognosis of elderly patients with chronic kidney disease was relatively dismal compared with those without a medical history of renal involvement.

In addition, one patient with a prior diagnosis of MN infected with SARS-CoV-2 following the administration of RTX. He achieved viral elimination within 3 weeks after anti-virus medications, and no serious adverse events occurred (Table 1, case 6).

## 3. New-Onset and Relapsed MN post COVID-19 Vaccination

### 3.1. Clinical Features and Follow-Up

#### 3.1.1. All Cases

Further, we conducted a literature search based on PubMed, EMBASE, Google Scholar, and Web of Science electronic database via the following keywords: (“membranous nephropathy” OR “proteinuria” OR “nephrotic syndrome”) AND (“SARS-CoV-2” OR “COVID-19” OR “2019-ncov” OR “novel coronavirus” OR “coronavirus”) AND (“vaccine” OR “vaccination”) to collect the clinical information of new-onset and relapsed MN post COVID-19 vaccination.

There were 37 cases reported before 6 September 2022 in 20 articles [4,15,16,17,18,19,20,21,22,23,24,25,26,27,28,29,30,31,32,33] (Table 2), including 20 (54.1%) cases with new diagnoses and 17 (45.9%) cases with relapsed or worsening symptoms. The median age of onset was 63.5 (22–84) years, and males accounted for 67.6% (25/37). mRNA vaccines were the principal type (30/38, 78.9%), followed by adenovirus vector vaccines (5/38, 13.2%). More than half of all patients were secondary to the second dose of vaccine within two weeks. The most frequent clinical presentation was edema. There were 10 cases (10/37, 27.0%) that suffered from AKI. However, AKI occurred in 4 out of five patients with MN secondary to SARS-CoV-2 infection. Among these 14 cases with available data of PLA2R staining, 11 presented with positive expression. In total, 20 of 26 (76.9%) cases showed an elevated level of PLA2R-Ab, in which 8 cases exceeded 50 RU/mL. Most of the cases (24/37, 64.9%) were given immunosuppressive therapies, and 12 cases were treated conservatively. A total of 24 cases responded to conservative, conventional immunosuppressive medications with or without RTX. All data could be acquired in Table 3.

#### 3.1.2. New-Onset MN

We collected 20 patients with new-onset MN post COVID-19 vaccination, and the median age of onset was 57 (22–82) years, of which 12 were males. mRNA vaccines were the leading type (15/21, 71.4%), usually occurring after the second dose of vaccine (10/20, 50.0%), with the most common onset time within two weeks (10/20, 50.0%). Edema and proteinuria were commonly observed in these cases. In total, 6 of 20 cases (30%) showed AKI. There were 7 cases associated with PLA2R, one case [4] with thrombospondin type-1 domain-containing 7A (THSD7A), and the other case [21] was diagnosed as neural epidermal growth factor-like 1 (NELL-1) related MN.

A total of 11 cases were treated with immunosuppressive drugs, 8 of which were given RTX, 4 patients achieved remission within the follow-up period, 1 case showed no response within 2 months, and 3 cases were lost to follow-up. Another 9 cases received conservative measures, 7 cases underwent remission, 1 case showed no response, and 1 case had no follow-up information. The median remission time was 41 (14–180) days. In general, the clinical treatment effect on COVID-19 vaccination-associated MN was worthy of being recognized. Table 2 and Table 3 illustrated the detailed data.

#### 3.1.3. Relapsed or Worsening MN

Overall, 17 cases showed worsening edema and proteinuria. Of the majority of enrolled patients, 76.5% (13/17), were males, with the median age of 65 (39–84) years. A total of 15 cases were associated with mRNA vaccines, in which Pfizer-BioNTech accounted for 86.7% (13/15). Two doses of vaccines were more likely to cause disease recurrence. Among these 17 patients, 8 patients relapsed within 2 weeks. In total, 14 of 15 cases (93.3%) were represented as an elevation of PLA2R-Ab. AKI was reported in 4 cases (4/17, 23.5%).

There were 13 cases treated with immunosuppressive medications, of which three cases responded to TAC, 2 to prednisone, and 1 patient using obinutuzumab had unclear prognostic information. A total of 7 cases received RTX, only one elderly patient [29] showed no remission in 4 months. Three cases received conservative treatment, only 1 case [31] showed improvement in proteinuria. The median remission time was 58 (30–180) days. In all relapsed cases, in 3 patients [29] using immunosuppressive medications during vaccination, edema occurred (Table 2 and Table 3).

### 3.2. Treatment and Prognosis

Generally, clinical cases of new-onset and relapsed MN associated with COVID-19 vaccination had an excellent prognosis. Some patients, especially new-onset patients, could achieve remission via conservative management. The median remission time was 30 (14–210) days. In contrast, conventional immunosuppressive drugs combined with RTX were required for relapsed patients, and the median remission time was 60 (21–180) days. It was widely accepted that PLA2R-Ab was a crucial clinical indicator for predicting the prognosis of MN [34]. Among these 20 cases of MN secondary to COVID-19 vaccination with positive expression of PLA2R-Ab, 14 had achieved remission in the follow-up period, and the median remission time was 60 (21–180) days (Table 2).

## 4. Discussions

### 4.1. Potential Mechanisms of MN post SARS-CoV-2 Infection

#### 4.1.1. Subepithelial Deposition of Viral Antigens

**Figure 1:**
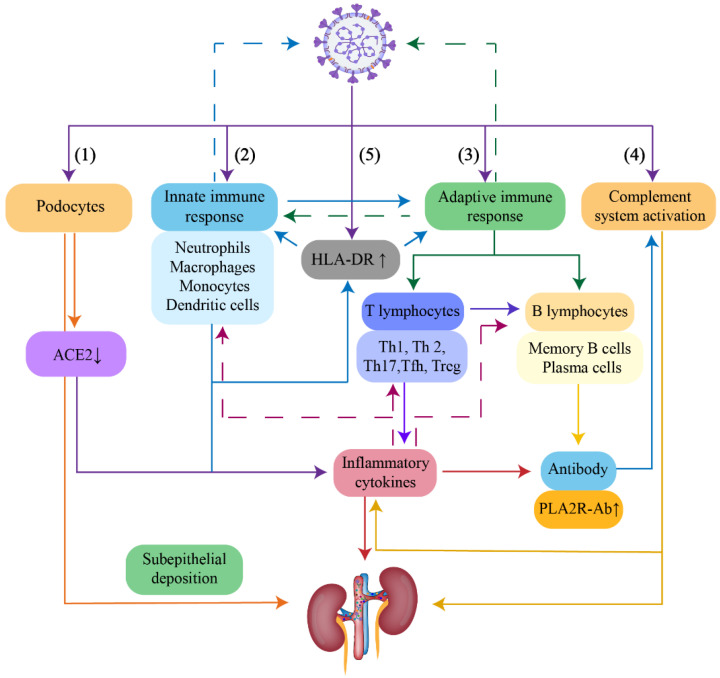
Potential mechanisms of MN post SARS-CoV-2 infection. (1) SARS-CoV-2 directly infected podocytes through binding to ACE2 on the surface of podocytes, which contributed to viral subepithelial deposition and resulted in functional ACE2 decreased, forming in situ immune complexes and promoting inflammatory response. (2) Viral infection initiated the innate immune response via recruiting inflammatory cells. Certain inflammatory cells probably functioned to express PLA2R. Once activated, these cells could release PLA2R antigen and stimulate B lymphocytes to produce PLA2R-Ab. (3) The activation of the innate and adaptive immune system promoted the release of inflammatory cytokines, which induced T and B lymphocyte differentiation, and the generation of antibodies. (4) SARS-CoV-2 activated the complement system, causing podocyte injury and further release of inflammatory cytokines. (5) Elevated levels of HLA-DR in monocytes promoted antigen presentation to the innate and adaptive immune system, leading to enhanced immune response.

Spike protein was the pivotal structure for SARS-CoV-2 to infect host cells through specifically recognizing ACE2 [35] and with the assistance of being cleaved by transmembrane protease serine 2 (TMPRSS2) [36,37]. Notably, both ACE2 and TMPRSS2 were also expressed on podocytes [38]. According to reports in the literature, SARS-CoV-2 viral particles were detected in podocytes of postmortem kidney samples in 26 patients with COVID-19 [39], indicating potential evidence that SARS-CoV-2 could directly invade into podocytes. Nevertheless, the process of viral infection on podocytes might contribute to the subepithelial deposition of viral antigens [40], thus forming in situ antigens to stimulate the production of corresponding antibodies, leading to the deposition of viral immune complexes in glomeruli (Figure 1).

#### 4.1.2. Massive Release of Cytokines

In COVID-19, viral infection in pulmonary epithelial cells triggered the recruitment of immune effector cells and released massive proinflammatory cytokines and chemokines [41,42], which subsequently advanced T lymphocyte differentiation. T helper (Th) 17 cells generated interleukin (IL)-17A, IL-17F, IL-22, and granulocyte-macrophage colony-stimulating factor, inducing the aggregation of inflammatory cells, such as neutrophils [43]. IL-4 produced by Th2 cells and IL-21 produced by follicular T helper (Tfh) cells contributed to B lymphocyte survival and proliferation as well as generated higher affinity to the IgG4 antibody [44]. Moreover, IL-4, IL-13, and IL-10 could promote the conversion of antibody category to IgG4 [44]. In addition, decreased levels of Th1 cells and regulatory T (Treg) cells secondary to SARS-CoV-2 infection destroyed immune tolerance [45].

In addition to acting as receptors, ACE2 also presented critical functions as a counter-regulatory enzyme to convert angiotensin II (Ang II) into Ang-(1–7), the latter of which performed attenuating inflammation effects [46]. Virus-occupied ACE2 might weaken their intrinsic function, which could enhance inflammatory response, neutrophils accumulation, and vascular permeability, and ultimately result in influenza-like symptoms, even severe acute respiratory distress syndrome among SARS-CoV-2 infected individuals [47], whereas in the kidneys, elevated immune response made it easier to develop glomerular diseases, such as MN (Figure 1).

#### 4.1.3. Speculation about PLA2R Antigen

Accumulating evidence has demonstrated that PLA2R, a pathogenic antigen of MN, was expressed not only in airway epithelial cells [48], neutrophils [49], and pulmonary macrophages [50], but also in podocytes. Once activated by foreign antigens such as SARS-CoV-2, these cells could secret extracellular vesicles containing PLA2R or cause the spatial release of PLA2R by generating extracellular traps, subsequently stimulating B lymphocytes to produce PLA2R-Ab [51]. In addition, the oxidation environment induced by inflammatory cytokines could bring about long-term expression of PLA2R pathogenic epitopes and enhance the capacity of binding to circulating antibodies [51] (Figure 1).

#### 4.1.4. Activation of the Complement System

The previous literature reported that the concentration of anti-SARS-CoV-2 immunoglobulin lacking glycan fucosylation was elevated in COVID-19 patients [52], which could help mannose-binding lectin to combine with aberrant glycans, thereby activating the complement system. The formed C5b-9 membrane attack complex on podocyte membranes participated in mediating the proteolysis of podocyte synaptophysin and NEPH1, resulting in the destruction of podocyte cytoskeleton [53,54] and eventually proteinuria (Figure 1).

#### 4.1.5. Elevated Expression of Human Leukocyte Antigen

Human leukocyte antigen (HLA) was expressed on the surface of immune cells and acted as presenters of epitopes to CD4^+^ T cells [55], indicating potent immunoregulatory properties. The present study demonstrated that activation of HLA-DR in circulating monocytes increased instantaneously in patients with SARS-CoV-2 infection [56], which could promote antigenic epitopes presentation to T lymphocytes and destroy immune tolerance (Figure 1).

### 4.2. Potential Mechanisms of MN post COVID-19 Vaccination

#### 4.2.1. Cross Immune Response

**Figure 2:**
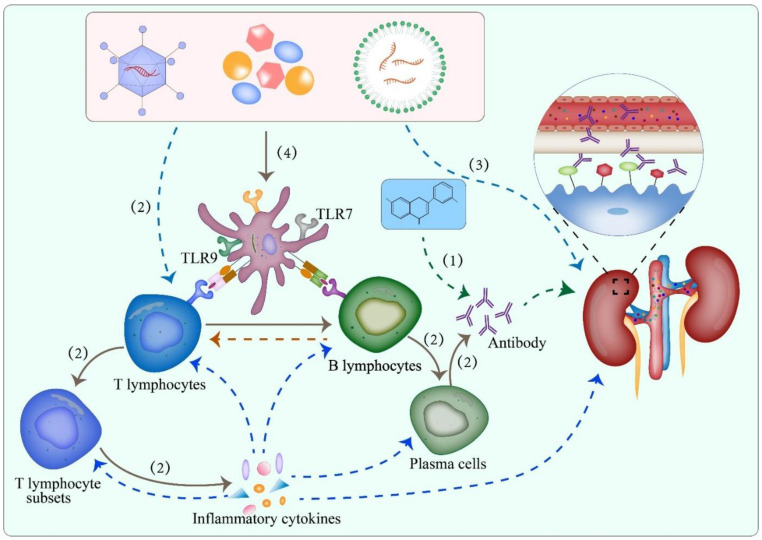
Potential mechanisms of MN post COVID-19 vaccination. (1) Spike or other structural proteins shared many amino acid sequences with human tissue proteins. Non-specific cross-immune response increased the risk of antibody binding to podocyte pathogenic antigens. (2) Vaccines activated immune effector cells, causing T lymphocyte differentiation and releasing massive inflammatory cytokines, which subsequently induced an enhanced immune response. (3) Vaccine acted as antigens, binding to native antibodies in vivo to form circulating immune complexes and deposited in the glomeruli. (4) Adjuvants provided pathogen-associated molecular patterns (PAMPs) and were recognized by toll-like receptors (TLRs) on the surface of antigen-presenting cells (APC) to elevate inflammatory response.

Some scholars have proven that amino acid sequence similarity between hepatitis B virus surface antigen and multiple sclerosis (MS) autoantigens might be part of the account of MS secondary to hepatitis B vaccination [57]. Recently, Vojdani et al. conducted an investigation aimed at studying the relationship between autoimmune target proteins and SARS-CoV-2 spike protein antibodies, the results of which proved that there were multiple tissue antigens that showed powerful reactions with the SARS-CoV-2 antibodies, such as transglutaminase 3, anti-extractable nuclear antigen, thyroid peroxidase, etc. [58], which indicated the fundamental role of cross-immune response in autoimmune diseases. Therefore, we proposed an underlying mechanism that podocyte surface-specific antigens might share similar amino acid sequences with spike protein or other components of SARS-CoV-2, which would be further supported along with the discovery of more pathogenic antigens on the surface of podocytes (Figure 2).

#### 4.2.2. Subepithelial Deposition of Circulating Immune Complexes

Proverbially, intramuscular vaccine components served as foreign substances to evoke the host’s immune response. As antigens, the vaccines promoted subepithelial deposition of circulating immune complexes in renal tissue via combining with native antibodies in vivo, which was plausible for explaining the MN secondary to influenza vaccines [59] (Figure 2).

#### 4.2.3. Enhanced Immune Response

Anti-SARS-CoV-2 neutralizing antibodies were of particular significance in evaluating protective immunity. For 250 patients with past-COVID-19, all moderate-severe patients and more than 80% of mild patients had positive antibodies [60]. Undeniably, vaccination was indeed an important initiative to enhance immune response. Compared with other types of COVID-19 vaccines, mRNA vaccines have been revealed to induce a more potent immune response. The rate of seroconversion was observed to increase five-fold from the baseline after the first dose of mRNA vaccines at two weeks [60]. Two doses of vaccines effectively induced antibody titers to exceed 300 U/mL and without evident decrease at 2 months [60]. Vaccines strengthened virus-specific responses and effectively activated T and B lymphocytes, accompanied by elevated generation of T cell inflammatory cytokines (e.g., interferon γ, tumor necrosis factor α, and IL-2, etc.) and higher levels of antibody titers, especially two doses of vaccines, including elderly individuals [61,62]. Due to the dose-dependent characteristics of COVID-19 vaccines, most adverse immune events typically occurred post the second dose [63] (Figure 2), which was consistent with our result. According to the clinical records we collected, only four new-onset patients had available data on antibody titers (case 2, 147.0 U/mL; case5, 32.8 U/mL; case19, 2334 U/mL; case 20, 1500 U/mL, Table 2). Consequently, further studies regarding the detection of antibody titers were required, which might contribute to clarifying the correlation between antibody level and disease onset.

#### 4.2.4. Adjuvants

The application of adjuvants achieved the possibility that small doses of vaccines could stimulate individuals to generate sufficient antibodies. Adjuvants, as antigens, could provide pathogen-associated molecular patterns and be recognized by toll-like receptors on the surface of antigen-presenting cells, activating downstream inflammatory signaling pathways and inducing an enhanced immune response [64]. Inactivated vaccines had decreased immunogenicity and usually required adjuvants, especially in elderly individuals considering immune senescence [65] (Figure 2).

### 4.3. Limitations

This article had several limitations. Most clinical cases were reported from single-case studies, and further research was needed to verify the causal relationship between MN and SARS-CoV-2 infection and COVID-19 vaccination. Moreover, there was the possibility that multiple cases were not reported, which might perplex us concerning the clinical characteristics and treatment response of MN associated with SARS-CoV-2 infection and COVID-19 vaccination. Furthermore, the pathogenesis involved in this review was based on our hypothesis, which demanded additional verification in the future. In addition, insufficient clinical information might lead to errors in data analysis.

## 5. Conclusions

In the era of the COVID-19 pandemic and the widespread requirement for vaccines, MN was an unavoidable but uncommon disease. Edema and proteinuria were the leading clinical manifestations. Overall, most cases had a good prognosis. Conservative and conventional immunosuppressive therapies with or without RTX promoted disease remission. Of note, it was not emphasized enough on the detection of antibody titers in currently published cases of MN post COVID-19 vaccination, which would be meaningful for assessing the potential association between antibody levels and disease onset. In addition, whether routine urine testing after vaccination contributed to the timely detection of the disease deserved more attention. In conclusion, further exploration was urgently needed to advance our knowledge of the incidence and recurrence rate, pathogenesis, treatment, and prognosis of MN post SARS-CoV-2 infection and COVID-19 vaccination.

## Figures and Tables

**Table 1:** Summary of published cases information of MN post SARS-CoV-2 infection.

Case	Age/Sex	Country	Medical History	Time after Diagnosis	Symptoms	Kidney Biopsy		Laboratory Characteristics before Treatment			Treatment	FU	Outcome	Others	Ref.
						PLA2R	VP	UPRO	ALB	PLA2R-Ab					
**New-Onset MN**															
1	70/F	America	HT, CAD, PVD, cervical carcinoma, GERD, HLD, obesity	UN	Cough, fever, dyspnea, edema, AKI	Neg	Neg	PCR:6.8 g/g	30 g/L	UN	No	35 d	R PCR:5–6g/g	-	[9]
2	72/M	America	HT, DM, HLD, gout, spinal stenosis, atrial fibrillation	UN	Cough, pleural effusion, edema	Pos	Neg	PCR:8.8 g/g	17 g/L	UN	TAC	18 d	UN	Repeated COVID-19 positive	[9]
3	81/M	Spanish	Prostate Ca, prediabetes, HLD, HT, CKD stage 3, AVS, CR	6 d	Fatigue, dyspnea, myalgia, sore throat, dry cough, poor appetite, loss of smell and taste, nausea, diarrhea, AKI, urinary incontinence, proteinuria	Neg	Neg	4.6 g/d	17 g/L	Neg	Lenzilumab, antibiotics, mPSL, heparin, dialysis	16 d	Death ALB:26 g/L when 7d on admission	Scr:3.7 mg/dL hematuria	[10]
4	29/M	South Asian	UN	4 W	Fever, myalgia, edema, AKI	Pos (weak)	Neg	8.7 g/d PCR:7.5 g/g	22 g/L	Pos	ACEI	3 Mo	NR PCR:11.9 g/g ALB:31 g/L	-	[11]
								PCR:9.2 g/g	23 g/L	UN	CTX, PSL	2 Mo	R PCR:4.9 g/g ALB:29 g/L		
5	71/M	America	HT, obesity, CKD	UN	Dyspnea, fever, cough, nausea, proteinuria, AKI	Neg	Neg	14.0 g/d	27 g/L	UN	Dialysis	80 d	NR Dialysis dependent	Scr:5.7 mg/dL	[12]
**RTX-treated MN infected SARS-CoV-2**															
6	48/M	Turkey	DM, MN	NA	Cough, fever, headache	UN	NA	UN	19 g/L	Pos	Oseltamivir, moxifloxacin, HCQ, azithromycin, lopinavir/ritonavir	3 W	R	Infection within 1 Mo post RTX treatment	[13]

Abbreviations: ACEI, angiotensin-converting-enzyme inhibitor; AKI, acute kidney injury; ALB, albumin; AVS, aortic valve stenosis; Ca, cancer; CAD, coronary artery disease; CKD, chronic kidney disease; CR, cervical radiculopathy; CTX, cyclophosphamide; d, day; DM, diabetes mellitus; F, female; FU, follow-up; GERD, gastroesophageal reflux disease; HCQ, hydroxychloroquine; HLD, hyperlipidemia; HT, hypertension; M, male; MN, membranous nephropathy; Mo, month; mPSL, methylprednisolone; NA, not applicable; Neg, negative; NR, no response; PCR, protein–creatinine ratio; PLA2R, phospholipase A2 receptor; PLA2R-Ab, phospholipase A2 receptor antibody; Pos, positive; PSL, prednisolone; PVD, peripheral vascular disease; R, response; RTX, rituximab; Scr, serum creatinine; TAC, tacrolimus; UN, unknown; UPRO, urine protein; VP, viral particles; W, week.

**Table 2:** Summary of published cases information of MN post COVID-19 vaccination.

Case	Age/Sex	Race/ Country	Medical History	Vaccine	Which Dose	Time	Symptoms	PLA2R	Baseline Scr	Laboratory Characteristics before Treatment				Treatment	FU	Outcome	Others	Ref.
										UPRO	ALB	Scr	PLA2R-Ab					
**New-Onset MN**																		
1	76/M	France	HT, UV-treated cutaneous mycosis fungoid	mRNA (Pfizer-BioNTech)	1	4 d	Edema	NA	Normal	PCR: 6.5 g/g	16 g/L	0.9 mg/dL	1:800	RASB	3 W	R PCR: 3.0 g/g ALB: 26 g/L	Hematuria	[15]
				mRNA (Moderna)	1	2 d	Edema, AKI	NA		PCR: 3.8 g/g	22 g/L	1.2 mg/dL	UN	RTX	2 Mo	R ALB: elevated		
2	70/M	Singapore	No	mRNA (Pfizer-BioNTech)	1	1 W, 1 d after 2nd dose	Edema, AKI	Neg	Normal	4.4 g/d	17 g/L	1.3 mg/dL	Neg	Irbesartan, furosemide, warfarin	2 Mo	NR	THSD7A-Ab: Pos COVID-19 antibody: 147.0 U/mL	[4]
3	56/M	America	HT	mRNA (Moderna)	1	1 Mo	Edema, AKI	Pos	Normal	1.7 g/L	22 g/L	14.0 mg/dL	Pos	PSL, amlodipine, clonidine, labetalol, RTX	1 Mo	R Renal function normalized	COVID-19 symptoms 6 Mo ago and edema 4 Mo ago	[16]
4	68/M	Germany	UN	mRNA (Pfizer-BioNTech)	1	7 d	Edema, AKI	Pos	UN	PCR: 19.0 g/g	29 g/L	UN	UN	Irbesartan	4 Mo	NR PCR: 14.4 g/g eGFR: decreased	-	[17]
										PCR: 14.0 g/g	UN	UN		RTX	2 Mo	R PCR: 6.0 g/g eGFR: elevated		
5	42/F	UN	UN	Adenovirus vector (AstraZeneca)	1	2 W	Edema, foamy urine, hair loss	UN	Normal	UN	16 g/L	0.8 mg/dL	Neg	GC, HCQ, MMF, diuretics	3 W	R Edema improved	ANA: 1:1280 COVID-19 antibody:32.8 U/mL	
6	68/M	White	UN	Adenovirus vector (Johnson & Johnson)	1	<4 W	AKI, CKD, NS	Pos	UN	0.6 g/d	32 g/L	3.3 mg/dL	UN	Diuretics	3 W	R UPRO: 0.4 g/d Scr: 2.7 mg/dL	-	[19]
7	57/F	China	No	Inactivated (Sinovac Life Sciences)	1	1 d	Edema	Pos	Normal	1.6 g/d	29 g/L	0.4 mg/dL	48 RU/mL	Losartan	4 W	R Edema improved	-	[20]
8	50/F	White	UN	mRNA (Pfizer-BioNTech)	2	4 W	Joint pain, proteinuria	UN	0.8 mg/dL	6.5 g/d	35 g/L	0.7 mg/dL	Neg	Conservative	2 Mo	R UPRO: 0.4 g/d ALB: 43 g/L	NELL-1-related, hematuria	[21]
9	82/F	Italy	No	mRNA (Pfizer-BioNTech)	2	88 d	NS	UN	UN	UN	UN	UN	Neg	GC	UN	UN	-	[22]
10	67/F	Italy	No	mRNA (Pfizer-BioNTech)	2	89 d	NS	UN	UN	UN	UN	UN	Neg	RTX	UN	UN	-	[22]
11	82/M	Italy	No	mRNA (Pfizer-BioNTech)	2	29 d	NS	UN	UN	UN	UN	UN	Pos	RTX	UN	UN	-	[22]
12	80/M	UN	CKD	UN	2	UN	Proteinuria, hematuria	Pos	UN	UN	UN	UN	UN	GC, RTX, CTX	UN	UN	Elevated p-ANCA, c-ANCA and MPO, positive of SSA-Ab	[23]
13	80/F	Italy	TP, HT, IH, AVB, hypothyroidism	mRNA (Pfizer-BioNTech)	2	2 W	Edema, AKI	UN	1.4 mg/dL	10.0 g/d	20 g/L	2.0 mg/dL	Pos	Steroids, CTX	6 Mo	R UPRO: 0.7 g/d, ALB: 40 g/L	FSGS, previously used steroids, hematuria	[24]
14	52/F	Italy	UN	mRNA (Pfizer-BioNTech)	2	49 d	Asthma, proteinuria	UN	Normal	3.4 g/d	UN	0.6 mg/dL	UN	RASB	41 d	R	-	[25]
15	UN	UN	UN	mRNA (UN)	UN	<3 W	UN	Pos	UN	PCR: 4.5–7.6 g/g	36 g/L	0.5–1.2mg/dL	UN	RASB	UN	UN	-	[26]
16	54/M	Asia	UN	mRNA (Moderna)	2	1 d	NS	Neg	UN	3+	34 g/L	1.3 mg/dL	UN	Steroid, RTX	8 W	NR UPRO: 3.5 g/d Scr: 0.9 mg/dL	ANA: Pos, ANCA: Pos, hematuria	[19]
17	47/M	Asia	UN	mRNA (Moderna)	2	6 d	NS	Neg	UN	2.7 g/d	23 g/L	0.7 mg/dL	UN	No	2 W	R UPRO: 2.7 g/d	Hematuria	[19]
18	22/M	Australia	Eczema, epilepsy	mRNA (Pfizer-BioNTech)	2	1 Mo	Edema, lethargy	Pos	Normal	ACR: 700.4 mg/mmol	8 g/L	0.7 mg/dL	118 RU/mL	Perindopril, frusemide anticoagulation	3 Mo	NR PCR: 1.1 g/mmol edema: worsened	-	[27]
														RTX	2 Mo	R PCR: 0.4 g/mmol		
19	32/M	India	UN	Adenovirus vector (AstraZeneca)	UN	14 d	NS, thrombosis	UN	UN	UN	UN	UN	UN	Conservative	UN	R	COVID-19 antibody:2334 U/mL	[28]
20	47/M	India	UN	Adenovirus vector (AstraZeneca)	UN	11 d	Proteinuria	UN	UN	UN	UN	UN	UN	ARB	UN	R	COVID-19 antibody:1500 U/mL	[28]
**Relapsed or Worsening MN**																		
1	77/F	America	MN	mRNA (Pfizer-BioNTech)	1	4 W	Edema	UN	0.8 mg/dL	PCR: 12.5 g/10 mmol	22 g/L	0.7 mg/dL	83 RU/mL	TAC	2 Mo	R	-	[29]
2	56/M	America	MN	mRNA (Pfizer-BioNTech)	1	2 W	Edema, fatigue	UN	1.5 mg/dL	3.4 g/d	32 g/L	1.5 mg/dL	30 RU/mL	Conservative	7 Mo	NR	RTX after 7 Mo, SARS-CoV-2 infected previously	[29]
3	65/F	UN	Systemic sarcoidosis, MN	Adenovirus vector (Johnson& Johnson)	1	5 Mo	Joint, skin, respiratory symptoms, AKI	Pos	UN	PCR: 3.4 g/g	UN	1.7 mg/dL	Pos	PSL	UN	R PCR: 1.8 g/g	-	[30]
4	67/M	Malaysia	MN	mRNA (Pfizer-BioNTech)	1	2 W	Proteinuria	UN	UN	5.3 g/d	UN	UN	42 RU/mL	Conservative	1 Mo	R UPRO: 1.6 g/d	CTX, steroids previously	[31]
5	66/F	Turkey	HT, DM, HLD, MN	Inactivated (Sinovac Life Sciences)	1	2 W	Edema, AKI	UN	Normal	PCR: 9.4 mg/mg	26 g/L	2.8 mg/dL	121 RU/mL	UN	UN	UN	Previously on steroids and CsA but off 7 Y	[32]
6	48/M	America	MN	mRNA (Pfizer-BioNTech)	1	2 W	Edema	UN	0.9 mg/dL	UN	25 g/L	1.3 mg/dL	155 RU/mL	RTX, CTX, PSL	3 Mo	R	RTX and TAC were using during vaccination	[29]
7	39/M	White	MN	mRNA (Pfizer-BioNTech)	2	1 W	Edema	Pos	0.9 mg/dL	8.7 g/d	20 g/L	1.1 mg/dL	UN	TAC	1 Mo	R UPRO: 5.7 g/d ALB: 29 g/L	Hematuria	[21]
8	70/M	White	MN	mRNA (Moderna)	2	4 W	Edema	Pos	1.7 mg/dL	16.6 g/d	27 g/L	2.1 mg/dL	UN	Obinutuzumab	UN	UN	-	[21]
9	80/M	America	MN	mRNA (Pfizer-BioNTech)	2	4 W	Edema	UN	1.2 mg/dL	5.0 g/d	26 g/L	1.3 mg/dL	916 RU/mL	RTX	4 Mo	NR	-	[29]
10	60/M	America	MN	mRNA (Pfizer-BioNTech)	2	6 W	Edema, dry mouth, skin rash, AKI	UN	1.4 mg/dL	5.0 g/d	17 g/L	1.9 mg/dL	27 RU/mL	RTX, CTX, PSL	3 Mo	R	TAC was using during vaccination	[29]
11	78/M	America	MN	mRNA (Pfizer-BioNTech)	2	1 W	Edema, HT	UN	1.6 mg/dL	PCR: 4.9 g/10mmol	34 g/L	1.9 mg/dL	Neg	PSL	1 Mo	R	-	[29]
12	48/M	America	MN	mRNA (Pfizer-BioNTech)	2	3 W	Edema	UN	1.4 mg/dL	PCR: 1.7 g/10mmol	31 g/L	1.4 mg/dL	204 RU/mL	Conservative	6 Mo	NR	RTX, CTX and PSL after 6 Mo	[29]
13	62/F	UN	Metastatic breast Ca, HT, HLD, MN	mRNA (Moderna)	2	2 W	Edema, dyspnea, proteinuria, AKI	Pos	UN	11.2 g/d	UN	1.6 mg/dL	787 RU/mL	Lisinopril, furosemide, RTX	UN	R PCR: 8.7 mg/g	-	[33]
14	84/M	America	MN	mRNA (Pfizer-BioNTech)	2	10 W	Edema, dyspnea	UN	1.5 mg/dL	3.0 g/d	33 g/L	1.5 mg/dL	Pos	TAC, PSL, RTX	4 Mo	R	SARS-CoV-2 infected previously, PSL and TAC were using during vaccination	[29]
15	39/M	America	MN	mRNA (Pfizer-BioNTech)	2	4 W	Fatigue	UN	1.2 mg/dL	PCR: 3.7 g/10mmol	18 g/L	1.4 mg/dL	40 RU/mL	RTX, CTX, PSL	2 Mo	R	-	[29]
16	75/M	America	MN	mRNA (Pfizer-BioNTech)	2	2 W	Edema, fatigue	UN	0.8 mg/dL	8.0 g/d	21 g/L	0.9 mg/dL	90 RU/mL	RTX, CTX, PSL	UN	UN	-	[29]
17	58/M	America	MN	mRNA (Pfizer-BioNTech)	2	3 W	Edema	UN	1.0 mg/dL	8.0 g/d	24 g/L	1.0 mg/dL	22 RU/mL	TAC	2 Mo	R	SARS-CoV-2 infected previously	[29]

Abbreviations: AKI, acute kidney injury; ALB, albumin; ANA, antinuclear antibody; ANCA, antineutrophil cytoplasmic antibody; ARB, angiotensin receptor blocker; AVB, atrial ventricular block; Ca, cancer; CKD, chronic kidney disease; CsA, cyclosporin A; CTX, cyclophosphamide; d, day; DM, diabetes mellitus; eGFR, estimated glomerular filtration rate; F, female; FSGS, focal segmental glomerular sclerosis; FU, follow-up; GC, glucocorticoid; HCQ, hydroxychloroquine; HLD, hyperlipidemia; HT, hypertension; IH, ischaemic heart; M, male; MMF, mycophenolate mofetil; MN, membranous nephropathy; Mo, month; MPO, myeloperoxidase; NA, not applicable; Neg, negative; NELL-1, neural epidermal growth factor-like 1; NR, no response; NS, nephrotic syndrome; PCR, protein–creatinine ratio; PLA2R, phospholipase A2 receptor; PLA2R-Ab, phospholipase A2 receptor-antibody; Pos, positive; PSL, prednisone; R, response; RASB, renin-angiotensin system blockade; RTX, Rituximab; Scr, serum creatinine; SSA, Sjogren syndrome antigen A; TAC, tacrolimus; THSD7A-Ab, thrombospondin type-1 domain-containing 7A-antibody; TP, thrombocytopenic purpura; UN, unknown; UPRO, urine protein; W, week; Y, year.

**Table 3:** Clinical characteristics of MN post COVID-19 vaccination.

Characteristics	New-Onset (*n* = 20)	Relapsed or Worsening (*n* = 17)	Total (*n* = 37)	*p*
Age (years)	57 (22–82)	65 (39–84)	63.5 (22–84)	0.657
Male sex, *n* (%)	12 (60.0)	13 (76.5)	25 (67.6)	0.393
Medical history, *n* (%)				-
Hypertension	3 (15.0)	2 (11.8)	5 (13.5)	-
Diabetes mellitus	0 (0.0)	1 (5.9)	1 (2.7)	-
Autoimmune disease	0 (0.0)	1 (5.9)	1 (2.7)	-
Vaccine type, *n* (%)				0.672
mRNA	15 (71.4) *	15 (88.2)	30 (78.9) *	-
Pfizer-BioNTech	10 (66.7)	13 (86.7)	23 (76.7)	-
Moderna	4 (26.7)	2 (13.3)	6 (20.0)	-
Inactivated	1 (4.8)	1 (5.9)	2 (5.3)	-
Sinovac Life Sciences	1 (100.0)	1 (100.0)	2 (100.0)	-
Adenovirus vector	4 (19.0)	1 (5.9)	5 (13.2)	-
AstraZeneca	3 (75.0)	0 (0.0)	3 (60.0)	-
Johnson & Johnson	1 (25.0)	1 (100.0)	2 (40.0)	-
Unknown	1 (4.8)	0 (0.0)	1 (2.6)	-
Which dose, *n* (%)				0.322
First dose	7 (35.0)	6 (35.3)	13 (35.1)	-
Second dose	10 (50.0)	11 (64.7)	21 (56.8)	-
Unknown	3 (15.0)	0 (0.0)	3 (8.1)	-
Onset time after vaccination, *n* (%)				0.713
No more than 2 weeks	10 (50.0)	8 (47.1)	18 (48.6)	-
2 weeks-4 weeks	4 (20.0)	6 (35.3)	10 (27.0)	-
Beyond 4 weeks	5 (25.0)	3 (17.6)	8 (21.6)	-
Unknown	1 (5.0)	0 (0.0)	1 (2.7)	-
Symptoms, *n* (%)				-
Edema	14 (70.0)	14 (82.4)	28 (75.7)	-
Proteinuria	12 (60.0)	2 (11.8)	14 (37.8)	-
Acute kidney injury	6 (30.0)	4 (23.5)	10 (27.0)	-
PLA2R stanning, *n* (%) **				0.607
Positive	7 (70.0)	4 (100.0)	11 (78.6)	-
Negative	3 (30.0)	0 (0.0)	3 (21.4)	-
PLA2R-Ab, *n* (%) **				0.065
Positive	6 (54.5)	14 (93.3)	20 (76.9)	-
Negative	5 (45.5)	1 (6.7)	6 (23.1)	-
Treatment, *n* (%)				0.117
Immunosuppressive therapy	11 (55.0)	13 (76.5)	24 (64.9)	-
Conservative medication	9 (45.0)	3 (17.6)	12 (32.4)	-
Unknown	0 (0.0)	1 (5.9)	1 (2.7)	-
Outcome, *n* (%)				0.795
Response	13 (65.0)	11 (64.7)	24 (64.9)	-
Not response	2 (10.0)	3 (17.6)	5 (13.5)	-
Unknown	5 (25.0)	3 (17.6)	8 (21.6)	-

* Data of one case was not available. ** In the population with known information, those without available data were not included.

## Data Availability

Not applicable.

## Abbreviations

ACE2: angiotensin-converting enzyme 2; ACEI, angiotensin-converting enzyme inhibitors; Ang II, angiotensin II; AKI, acute kidney disease; COVID-19, coronavirus disease 2019; CTX, cyclophosphamide; IL, interleukin; MN, membranous nephropathy; MS, multiple sclerosis; NELL-1, neural epidermal growth factor-like 1; PLA2R, phospholipase A2 receptor; PLA2R-Ab, phospholipase A2 receptor antibody; RTX, rituximab; SARS-CoV-2, severe acute respiratory syndrome coronavirus 2; TAC, tacrolimus; Tfh, follicular T helper; Th, T helper; THSD7A, thrombospondin type-1 domain-containing 7A; TMPRSS2, transmembrane protease serine 2; Treg, regulatory T.
